# A Tool to Assess the Trustworthiness of Evidence-Based Point-of-Care Information for Health Care Professionals (CAPOCI): Design and Validation Study

**DOI:** 10.2196/27174

**Published:** 2021-10-05

**Authors:** Gerlinde Lenaerts, Geertruida E Bekkering, Martine Goossens, Leen De Coninck, Nicolas Delvaux, Sam Cordyn, Jef Adriaenssens, Bert Aertgeerts, Patrik Vankrunkelsven

**Affiliations:** 1 Belgian Centre for Evidence-Based Medicine Leuven Belgium; 2 Cochrane Belgium Leuven Belgium; 3 Department of Public Health and Primary Care KU Leuven Leuven Belgium; 4 Federation of the White and Yellow Cross of Flanders Brussels Belgium; 5 Belgian Health Care Knowledge Centre Brussels Belgium

**Keywords:** evidence-based medicine, evidence-based practice, point-of-care systems, health care quality, information science, practice guidelines as a topic

## Abstract

**Background:**

User-friendly information at the point of care for health care professionals should be well structured, rapidly accessible, comprehensive, and trustworthy. The reliability of information and the associated methodological process must be clear. There is no standard tool to evaluate the trustworthiness of such point-of-care (POC) information.

**Objective:**

We aim to develop and validate a new tool for assessment of trustworthiness of evidence-based POC resources to enhance the quality of POC resources and facilitate evidence-based practice.

**Methods:**

We designed the Critical Appraisal of Point-of-Care Information (CAPOCI) tool based on the criteria important for assessment of trustworthiness of POC information, reported in a previously published review. A group of health care professionals and methodologists (the authors of this paper) defined criteria for the CAPOCI tool in an iterative process of discussion and pilot testing until consensus was reached. In the next step, all criteria were subject to content validation with a Delphi study. We invited an international panel of 10 experts to rate their agreement with the relevance and wording of the criteria and to give feedback. Consensus was reached when 70% of the experts agreed. When no consensus was reached, we reformulated the criteria based on the experts’ comments for a next round of the Delphi study. This process was repeated until consensus was reached for each criterion. In a last step, the interrater reliability of the CAPOCI tool was calculated with a 2-tailed Kendall tau correlation coefficient to quantify the agreement between 2 users who piloted the CAPOCI tool on 5 POC resources. Two scoring systems were tested: a 3-point ordinal scale and a 7-point Likert scale.

**Results:**

After validation, the CAPOCI tool was designed with 11 criteria that focused on methodological quality and author-related information. The criteria assess authorship, literature search, use of preappraised evidence, critical appraisal of evidence, expert opinions, peer review, timeliness and updating, conflict of interest, and commercial support. Interrater agreement showed substantial agreement between 2 users for scoring with the 3-point ordinal scale (τ=.621, *P*<.01) and scoring with the 7-point Likert scale (τ=.677, *P*<.01).

**Conclusions:**

The CAPOCI tool may support validation teams in the assessment of trustworthiness of POC resources. It may also provide guidance for producers of POC resources.

## Introduction

Evidence-based medicine (EBM) aims to integrate the experience of the health care professional, the values of the patient, and the best available scientific information to guide clinical decision making. Back in 1996, Sackett defined EBM as “the conscientious, explicit, and judicious use of current best evidence in making decisions about the care of individual patients.” The practice of EBM means integrating individual clinical expertise with the best available external clinical evidence from systematic research [1,2]. However, keeping up to date with the best available evidence is time-consuming, and the format of a systematic review is impractical for quickly answering clinical questions [3]. As more and more health professions worldwide adopted the EBM concept, the name shifted in the last decade to evidence-based practice (EBP). Nowadays, clinical guidelines are the gold standard in EBP to guide the clinical decision process, but for many health problems and health care professions, there are no guidelines available [4]. Technological progress allows rapid and easy access to an enormous amount of information, but the trustworthiness is often unclear. Point-of-care (POC) information is defined as high-quality information needed by health care professionals when they interact with the patient; this information should be well structured, quick and easily accessible and, most importantly, relevant and reliable [5,6]. Many authors have highlighted the importance of trustworthiness or quality of web-based POC information [7-12]. For systematic reviews and clinical guidelines, well-developed critical appraisal tools are available (eg, AMSTAR [A Measurement Tool to Assess Systematic Reviews] [13,14] and AGREE II [Appraisal of Guidelines for Research and Evaluation II] [15]). However, these tools are not appropriate for the evaluation of POC information.

Trustworthy POC information sources for health care professionals require a robust methodological process for searching, appraisal, and synthesis of the best available evidence in a reproducible way. Health information for professionals differs at this point from health information for the lay public, and consequently the initiatives to evaluate health information for patients (eg, e-Health Code of Ethics [16], Health on the Net [17], Journal of the American Medical Association [18], and DISCERN [19]) are not suitable for the evaluation of POC information for professionals. A standard tool to evaluate the trustworthiness of POC information that is not a guideline is therefore essential.

This study built on the results of a systematic review [20] that searched for existing tools to assess the trustworthiness of POC information. The content of existing tools was analyzed, and the tools were examined for validity and reliability. However, a tool that is complete, usable, and validated could not be found. Therefore, the aim of this study was to develop a new tool for assessment of trustworthiness of evidence-based POC resources to enhance the quality of POC resources and support health care professionals to have access to reliable EBP information. This paper describes the development process and associated validity of this new tool, which we named the Critical Appraisal of Point of Care Information (CAPOCI) tool.

## Methods

### Systematic Review

In a first step, a systematic review was performed to search for already existing tools to assess trustworthiness of POC resources. This systematic review has been published as a separate paper [20]. We aimed to describe and analyze the content of these tools by documenting the general characteristics, purpose for which a tool was developed, and criteria and scoring systems that were used. We also checked whether the included tools were examined for validity and reliability. Seventeen tools were included in the review. The tools encompassed a variety of criteria important for assessment of trustworthiness of POC information. Only two tools were assessed for both reliability and validity, but they lacked some essential criteria for assessment of trustworthiness of health care information for use at the point of care, pointing to the need to develop a new tool.

### Formulation of Criteria for the CAPOCI Tool

In a second step, two methodologists (GB and GL) started from the results of the systematic review to derive relevant criteria for the CAPOCI tool. They related the relevance of criteria to frequency of occurrence in the existing tools and contribution of the criteria to the trustworthiness of POC information according to the reviewers. A working group of health care professionals and methodologists (ie, the authors of this paper) then discussed the relevance, applicability, and accurate wording of the different criteria. The further refinement of the items for the CAPOCI tool was the result of an iterative process of discussion and pilot testing until consensus was reached for all criteria, resulting in a tool with 9 criteria.

### Content Validation

The content validity of the CAPOCI tool was tested on an international panel of experts (Multimedia Appendix 1) with the RAND modified Delphi method [21]. Panel members were selected based on their authorship of publications in the domain and their affiliation to relevant institutions. All panel members received a payment of €250 (US $306) for their time investment. The whole process was in writing and anonymous, and only the study coordinator (GL) knew the identity of the panel members.

All panel members received a study protocol and an electronic questionnaire. They were asked to rate the relevance and wording (accuracy and correctness) of the different CAPOCI criteria. The panel members rated their agreement with each item as strongly agree, agree, disagree, or strongly disagree. If the answer was disagree or strongly disagree, an explanation was required.

**Figure 1:**
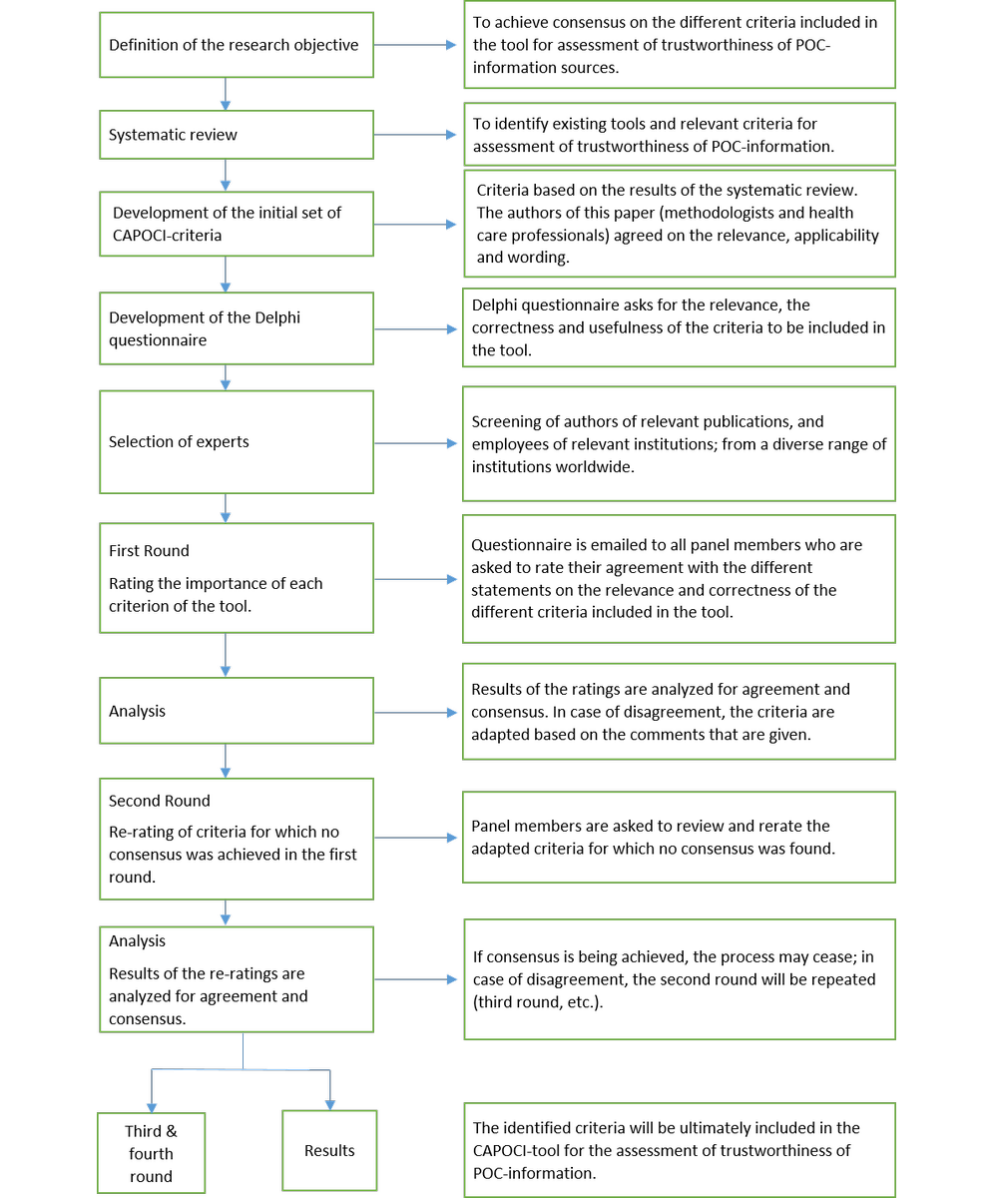
Overview of the development process of the Critical Appraisal of Point of Care Information (CAPOCI) tool. POC: point-of-care.

The questionnaires were returned to the study coordinator. Consensus on a criterion was achieved when 70% of the panelists rated a criterion as strongly agree or agree. Based on the justification formulated in case of disagreement, the criterion was reformulated and again presented to all panel members in a next round. The process was repeated until consensus was reached for all criteria. Figure 1 shows an overview of the development process of the CAPOCI tool.

### Scoring Methods for the CAPOCI Tool

The results of the systematic review did not identify the most appropriate scoring system for the CAPOCI tool. Therefore, we decided to test 2 scoring systems. The first scoring system rated each criterion on a 3-point ordinal scale with the following possible ratings: (1) fulfilled, (2) minor remark, and (3) major remark. The conditions for awarding one of the 3 ratings were discussed and defined by the authors of this paper. The second scoring system rated each criterion on a 7-point Likert scale, where a score of 1 indicated fully disagree and a score of 7 indicated fully agree.

### Interrater Reliability And Statistical Analysis

The CAPOCI tool was tested for interrater reliability. To this end, two methodologists (GB and GL) assessed 5 POC resources used in Belgian health care with the CAPOCI tool, using the 2 scoring systems described above.

The statistical analyses were performed using SPSS (version 24, IBM Corp). A 2-tailed Kendall tau correlation coefficient was calculated to quantify the agreement between the 2 reviewers for the entire CAPOCI tool per scoring system. Level of significance was set at .01. In addition, a descriptive analysis was performed for each criterion separately. We counted how often a criterion was scored differently by the 2 reviewers for the 5 evaluated POC resources. For scoring with the Likert scale, a difference in score of more than 1 was considered a different score. The reason for interrater disagreement was documented.

## Results

### Delphi Study

Based on the results of the systematic review, the CAPOCI tool was initially defined with 9 criteria. These 9 criteria were then tested in the Delphi study. Ten experts from a diverse range of organizations and institutions reputable in EBM worldwide participated (see Multimedia Appendix 1). One expert dropped out after the first round.

The 9 criteria were assessed as relevant by all the experts after the first round, but refinement of the wording was needed. After 4 rounds of the Delphi study, a final version of the CAPOCI tool was developed including 11 criteria, where the criteria related to authorship and experience of authors, literature search and surveillance were split up. Table 1 summarizes the CAPOCI criteria as defined by the expert panel, together with the conditions for rating a criterion as fulfilled, minor remark, or major remark.

**Table 1:** The criteria of the CAPOCI tool and the conditions for scoring on a 3-point ordinal scale.

CAPOCI^a^ criteria	Conditions to rate as fulfilled	Conditions to rate as minor remark	Conditions to rate as major remark
1. Authorship: The authors must be referenced on the website but do not need to be identified for each individual topic (clicking and searching may be necessary).	Name and affiliations of all authors are mentioned.	Only a general description is available (eg, of the editorial board).	There is no information available on the authors.
2. Expertise of the authors: The author team is qualified in the specific domain and can demonstrate their expertise on request.	The expertise of the author team is demonstrated.	The expertise of the author team is unclear.	There is no information available on the expertise of the author team.
3a. Literature search and surveillance: A systematic search strategy was used to search for source information.	A systematic search strategy has been used to search for source information. This search strategy is described in detail in the EBP^b^ source.	The description is not sufficiently detailed to be able to assess; there are inaccuracies in the methodological process.	Literature search seems to be implemented, but there is no description of the process or there is no information on how the literature search was done.
3b. Literature search and surveillance: Systematic methods were used for selection of the evidence from the search.	Systematic methods have been used to select the evidence from the results of the literature search. These methods are described in detail.	The description is not sufficiently detailed to be able to assess; there are inaccuracies in the methodological process.	A systematic selection process seems implemented, but there is no description of the process or there is no information on how this selection was done.
4. Critical appraisal of the evidence: A critical appraisal has been implemented to assess the validity of the evidence used. The critical appraisal must be scientifically robust and transparent. The critical appraisal assessment has informed the interpretation of the evidence.	An adequate critical assessment of the quality of scientific evidence has been performed, and the procedure has been described in a transparent way. The critical assessment serves as a basis for the interpretation of the evidence.	The description is not sufficiently detailed to be able to assess; there are inaccuracies in the methodological process.	It is unclear whether a critical assessment of study data has taken place.
5. Use of the best available evidence: The content of the EBP source should be based on the best available evidence, specific to the clinical question. Well-designed and conducted evidence synthesis documents, when available, are preferred above primary studies.	The content of the EBP source is based on the best available evidence, specific to the clinical question. If available, well-designed and conducted evidence synthesis documents are preferred over primary studies.	The description is not sufficiently detailed to be able to assess; there are inaccuracies in the methodological process.	It is unclear whether the authors prioritize evidence synthesis documents over primary studies.
6. Citation of expert opinions: When expert opinions are cited, this must be clearly indicated in order to distinguish it from empirical evidence. Experts should be listed along with their professional designation, organization, and a conflicts of interest statement.	It is clearly stated when expert opinions are cited to distinguish it from empirical evidence. There is a description of the expertise of the experts, along with their professional affiliations, including a declaration of possible conflicts of interest.	The description is not sufficiently detailed to be able to assess. The expertise of the experts is unclear or the affiliations and declaration of conflicts of interest are lacking.	It is unclear whether expert opinions are cited or the distinction between expert opinion and empirical evidence is unclear.
7. Review process: The scientific quality and the clinical applicability of the EBP source is assessed by peer reviewers.	There is a detailed description of the review process of the scientific quality and clinical applicability of the EBP source.	Only a general description of the review process is available (eg, “information was reviewed by external reviewers”).	There is no information available about the review process.
8. Timeliness and updating: The frequency of updates is determined by the speed of developments in the field and is documented in the methodology. The content of the EBP source is checked and updated when new information is available. The date of first publication, date of the last update, and data on the next planned update are clearly displayed in the EBP source.	The EBP source is frequently updated in accordance with the developments in the field. The frequency of the updates is documented in the methodology. The date of first publication and last update can be found in the source, as well as information on the next planned update.	Updates are performed but not sufficiently frequently, which means that the content may be out of date.	Insufficient information about updates; date of last update not displayed.
9. Conflicts of interest: There is a formal policy on declaring and managing financial and nonfinancial conflicts of interest of the authors and other stakeholders. Possible conflicts of interest are reported.	Procedure for conflicts of interest has been implemented and documented (conflicts of interest should not be explicitly stated on the website, but the information must be able to be submitted to the assessor).	Conflict of interest procedure seems implemented but not reported.	No information about conflict of interest procedure available (conflicts of interest are not checked or reported).
10. Commercial support: It is clearly described to what extent commercial support was accepted for developing the content of the EBP source. The financier has no substantive input and therefore no influence on the result or the content of the EBP source. When advertisements on websites are a source of income, this must be clearly stated on the site. A short description of the advertising policy is published on the site. Advertisements and other promotional material must be presented in such a way that visitors can clearly distinguish between editorial content.	If commercial support is accepted, this is clearly and publicly announced and there is no influence of the financier on the content or the result of the EBP source.	Not applicable.	There is insufficient information to judge.

^a^CAPOCI: Critical Appraisal of Point of Care Information.

^b^EBP: evidence-based practice.

### Interrater Reliability

Assessment of 5 POC resources with the CAPOCI tool (Multimedia Appendix 2) showed a substantial agreement between the reviewers (GB and GL) of .621 (*P*<.01) for scoring with the 3-point ordinal scale and .677 (*P*<.01) for scoring with the 7-point Likert scale, as calculated by the Kendall tau correlation coefficient.

The descriptive analysis of agreement between the reviewers for each criterion separately showed that both scoring systems lead to similar results (Table 2). There were no interrater differences in the scoring of criterion 5 (use of the best available evidence) for the 5 evaluated POC resources. For the other criteria, the most common reason for disagreement was a difference in rigor of application of the criterion by the 2 reviewers. For the scoring of criterion 6 (citation of expert opinion), the information found in the POC resources was differently interpreted by both reviewers, leading to a different score. Criterion 8 (timeliness and updating) and 9 (conflict of interest) were once scored differently because the necessary information was not always retrieved in the POC resource by both reviewers.

**Table 2:** Descriptive analysis of interrater agreement between 2 reviewers using the CAPOCI criteria on 5 POC resources. Scoring was done on a 3-point scale (fulfilled, minor remark, major remark) and on a 7-point Likert scale.

CAPOCI^a^ tool	Scoring with 3-point scale; number of interrater disagreements (/5)	Scoring with 7-point Likert scale; number of interrater disagreements (>1) (/5)	Reason for interrater disagreement		
			Difference in rigor of application	The information in the POC^b^ resource was differently interpreted by the reviewers	The information was found/not found in the POC resource by the reviewers
Criterion 1	1	0	✓	—^c^	—
Criterion 2	1	1	✓	—	—
Criterion 3a	1	1	✓	—	—
Criterion 3b	1	2	✓	—	—
Criterion 4	1	1	✓	—	—
Criterion 5	0	0	—	—	—
Criterion 6	2	1	—	✓	—
Criterion 7	2	2	✓	—	—
Criterion 8	1	2	✓	—	✓
Criterion 9	1	1	—	—	✓
Criteron 10	1	1	✓	—	—

^a^CAPOCI: Critical Appraisal of Point of Care Information.

^b^POC: point-of-care.

^c^Not applicable.

## Discussion

### Principal Findings

We developed a uniform, comprehensive, and validated tool specifically designed for the evaluation of POC information. The CAPOCI tool allows the systematic evaluation of the trustworthiness of POC resources, including the methodological process of searching, appraising, and synthesizing the best available evidence for a specific health topic.

### Content of the CAPOCI Tool

Our systematic review showed that items for assessing POC resources could be divided into 4 main domains: author-related information, criteria related to evidence-based methodology, criteria related to website quality, and criteria related to website design and usability [20]. The rigor and specificity with which each of these domains should be evaluated is an interesting discussion. For the development of the CAPOCI tool, we focused on the first 2 domains as these directly relate to the trustworthiness of the information. The tool can be used for different types of POC resources (eg, interactive websites as well as online PDFs). Website quality and design and usability of web-based POC resources can be evaluated by existing tools for assessment of readability [22], design evaluation, and accessible health information contents [23,24].

The rigorous reporting of author-related information is a first important point when we focus on trustworthiness of (web-based) POC information. In this internet era, where personal opinions are easily expressed, it is crucial to have one or more authors who take responsibility for the content of a POC resource. Clarity and transparency about authorship must always go hand in hand with transparent and consistent reporting of conflicts of interest. Users of EBP resources need to be sure that the provided information reflects the best evidence and potential influence by competing interests has been minimized. The disclosure of conflicts of interests in guideline development has received much attention after reports on undisclosed financial conflicts of interest in guideline panels [25]. In response to this, the American College of Physicians Clinical Guidelines Committee recently published their policy for disclosure of interests and management of conflicts of interest in clinical guidelines [26] and the Cochrane Collaboration announced a new conflict of interest policy in October 2020 to strengthen user confidence [27].

To fulfill the claim of being evidence-based, a POC resource should be built on a robust methodological process. First, an explicit methodology for literature search and surveillance helps to avoid biases. A literature search should be systematic and well-documented for transparency and auditability. However, a systematic search with nonsystematic article selection can negate the effort for systematic search and surveillance. Therefore, a systematic strategy for article selection should be worked out as well, including a clear definition of inclusion and exclusion criteria for the selection of source information. These are basic principles of comprehensiveness in literature search that are important and widely adopted for guideline development [28], but comprehensiveness is also essential for other POC resources.

Once the information is selected, it should be critically appraised to assess the validity of the evidence it provides and to prevent inclusion of biased results. Some sources such as Cochrane systematic reviews are conducted following a strict protocol, and therefore they can be considered as trustworthy. But there are an increasing number of systematic reviews available in which the quality is often suboptimal [29,30]. When a POC resource is derived from a clinical practice guideline, the guideline should be critically appraised preferably with the AGREE II instrument [15]. Despite international efforts for quality standards of guidelines, development processes of guidelines still vary substantially, and many guidelines do not meet basic quality criteria [31].

When the criteria for literature and surveillance and critical appraisal of evidence are met, the criterion concerning the use of best available evidence will probably be fulfilled as well. We added this criterion to the tool to clarify that the content production process of a POC resource should be based preferentially on well-designed and conducted evidence synthesis documents rather than single studies. The best available evidence can be explained in different ways. The 6S pyramidal model of Haynes is often used as a conceptual framework for searching information resources for EBM [32], suggesting that the best available evidence is always higher up in the pyramid. The 6S model was later reworked to the evidence-based health care pyramid 5.0 for accessing preappraised evidence and guidance [33]. This pyramidal model adds systematically derived recommendations as a major type of information and simpliﬁes the overall framework to 5 major layers of information types. Although these models give a good perspective on the difference in information types for EBM while focusing on preappraisal of information, it might be rather arbitrary since some resources may overflow between layers and may be difficult to attribute to one specific layer [6]. Furthermore, the best available evidence is also related to the clinical question to which the POC resource wants to provide an answer and the associated ideal study design. In epidemiological studies, the best available evidence will be observational data; in intervention studies, the best available evidence will be experimental data; and in studies of human experience, the best available evidence will be qualitative data. Levels of evidence frameworks such as the Oxford Centre for Evidence-Based Medicine table of evidence [34] are useful in the process of finding the appropriate evidence for a specific research question, but these frameworks predominantly address questions related to quantitative studies, while qualitative research is sometimes the best and most appropriate type of information to inform policy and planning decisions. Therefore, the framework with most relevance to the stakeholder group and most appropriate for the clinical question that is being asked should be considered when deciding on the best available evidence for the content of a POC resource.

For some clinical questions concerning rare diseases, best practice guidance, or treatments that are strongly context-dependent (eg, resistance for antibiotics), the evidence may be limited. In these situations, obtaining evidence from experts can be efficient, and experts may be the only or main source of evidence. However, the use of expert opinion should be clearly distinguished from empirical data in a POC resource [35]. To this end, the use of in-text referencing to published study data and clear statements on the use of expert opinion will contribute to more transparency and add to the trustworthiness of the POC resource.

Peer review, procedures for updating, and clear policies on conflict of interest and commercial support are basic principles for EBP that are present in all critical appraisal tools for clinical trials, systematic reviews, or guidelines [13,14,36]. Because of the POC aspect, a peer-review policy of a POC resource should not only address the scientific quality but also the clinical applicability of the information. To increase the transparency and auditability of the POC resource, the review process should be documented. For the same reason, procedures for updating should be documented. Systematic searches for new inputs should be executed with a frequency appropriate for the developments in the field of interest. An update should also be based upon either higher level of evidence or higher quality evidence; there is no point in adding new evidence if it is of lower quality or does not add empirical rigor.

POC resources are often web-based, which makes them very interesting for biomedical companies for online advertising. It is possible that a POC resource depends on commercial funding. In that case, a strict policy that ensures that the funder or advertiser has no influence on the content of the POC resource is mandatory. The distinction between content and advertising must always be very clear to the user.

### Scoring Systems

We tested 2 scoring systems for the CAPOCI tool, both showing good reliability. The purpose of the evaluation of a POC resource can be a determining factor in the choice of scoring system. The 7-point Likert scale allows more nuance. It is also used in the AGREE II instrument and therefore probably familiar for users. It allows for a quantitative comparison when rating and comparing different POC sources.

The 3-point scale may be better suited when different reviewers must formulate a final judgement on a POC resource (eg, for granting a quality label) in consensus. The predefined conditions for the 3 categories also allow structured feedback to developers for POC resources on the criteria where additional information is required.

### Use of the CAPOCI Tool

The CAPOCI tool is valid and reliable instrument that can be used by health care providers, researchers, and decision and policy makers for the evaluation of the trustworthiness of POC resources. Developers of POC resources can also use the tool as a guide in the development process.

Although the results of the interrater variability tests showed substantial agreement between reviewers for the CAPOCI tool, the descriptive analysis showed a difference in rigor of application between reviewers for 8 of the 11 criteria. In addition, different reviewers might interpret information in the POC resource differently, and the necessary information for evaluation was sometimes not retrieved. Therefore, a robust use of the CAPOCI tool includes an evaluation by at least 2 independent reviewers who reach a joint final judgment after discussion in order to reduce subjectivity and inaccuracy in the evaluation. Furthermore, reviewers should have experience with EBP methodology or should be trained by an experienced methodologist. Pilot testing is recommended to mitigate differences in rigor of application between reviewers and can be part of training for the good use of the CAPOCI tool.

### Strengths and Limitations

The CAPOCI tool is based on a rigorous development process starting from a systematic review to consider and cover all possible criteria for the assessment of trustworthiness of POC information. The author group who defined the initial CAPOCI criteria consisted of methodologists and health care professionals, each with broad experience in guideline development and/or guideline validation.

Of all POC tools that were previously analyzed in our systematic review, only 2 were validated and tested for reliability [20]. However, these are essential requirements for tools used for evaluation in the context of EBP that were adopted in the CAPOCI tool.

Reliability testing was performed by reviewers who were involved in the development of the tool, which might have influenced the results. We will use this tool among different methodologists at our institute. The criteria and scoring methods will be refined where needed based on the experience and feedback of the users. Adding examples to the different criteria may also contribute to a better understanding and ease of use for less experienced users. Furthermore, content validation was done with a panel of 10 experts and reliability testing with only 2 reviewers, which can be considered as a limitation of this study. Using more reviewers or a more extended expert panel would add to the quality of validity and reliability testing.

### Conclusion

With the development of the CAPOCI tool, we filled a gap in the evaluation of POC information. The CAPOCI tool facilitates the assessment of trustworthiness in POC resources. It may also provide guidance for producers of POC resources. Wide use of the CAPOCI tool may improve the quality and reliability of POC resources over time and may take EBP in daily practice to a higher level.

## Appendix

Multimedia Appendix 1 Members of the international panel of experts who participated in the Delphi study.

Multimedia Appendix 2 Scoring of five point-of-care resources with the Critical Appraisal of Point of Care Information tool.

## Abbreviations

AGREE II: Appraisal of Guidelines for Research and Evaluation II
AMSTAR: A Measurement Tool to Assess Systematic Reviews
CAPOCI: Critical Appraisal of Point of Care Information
EBM: evidence-based medicine
EBP: evidence-based practice
POC: point-of-care
