# Supplementary material for: A Tool to Assess the Trustworthiness of Evidence-Based Point-of-Care Information for Health Care Professionals (CAPOCI): Design and Validation Study
Source: J Med Internet Res. 2021 Oct 5;23(10):e27174. doi: 10.2196/27174 (PMC8527381; doi:10.2196/27174)
Supplement: Multimedia Appendix 1 [file jmir_v23i10e27174_app1.docx]

Appendix 1

Members of the international panel of experts, who participated in the Delphi study

Brian Alper, MD; Chief Medical Knowledge Officer, EBSCO Information Services, Ipswitch, United States.

Rita Banzi, PhD; Center for Health Regulatory Policies, Istituto di Ricerche Farmacologiche Mario Negri IRCCS, Milan, Italy

Matteo Capobussi, MD; Department of Biomedical Sciences for Health, University of Milan, Italy

Deborah Jane Collis, BSc MSc MPHe, Associate Director: System Engagement, National Institute for Health and Care Excellence (NICE), University of Manchester, United Kingdom

Anna Gagliardi, PhD; Institute of Health Policy, Management and Evaluation, Toronto University, Canada.

Rosella Hermens, MD, PhD; Radboud Institute for Health Science, Nijmegen, The Netherlands

Craig Lockwood, RN, PhD; Director Implementation Science, Joanna Briggs Institute; Adjunct Associate Professor Queens University School of Nursing, The University of Adelaide, Australia

Lorenzo Moja, MD, PhD; Department of Health Products Policy and Standards, World Health Organization

Zachary Munn, PhD; Director JBI Transfer Science, Director JBI Adelaide **GRADE** Centre, Vice-Chair Guidelines International Network (G-I-N); Joanna Briggs Institute, The University of Adelaide, Australia

Kees Van Boven, MD, PhD; Radboud University, Departement of Primary and Community Care, Nijmegen, The Netherlands
